# Accessing primary care following the Affordable Care Act: a qualitative study of low-income women’s experiences in urban California

**DOI:** 10.1186/s12913-025-13853-9

**Published:** 2026-02-04

**Authors:** Allison Gilchrist, Paula Holland, Faraz Ahmed

**Affiliations:** 1 Division of Health Research, Faculty of Health and Medicine, Lancaster University, Bailrigg, Lancaster, LA1 4YW UK; 2 School of Nursing, College of Health & Social Services, San Francisco State University, Burk Hall, 1600 Holloway Avenue, San Francisco, CA 94132 US

**Keywords:** Medicaid expansion, Affordable Care Act, Primary healthcare, Low-income women, Medically-Underserved populations, Qualitative research, Health services accessibility, Health policy, Social determinants of health, California

## Abstract

**Background:**

The 2010 Affordable Care Act (ACA) led to Medicaid expansion, which expanded eligibility to low-income individuals below 138% of the federal poverty level in 41 states and Washington, DC. In California, over one-third of state residents are covered by Medicaid (Medi-Cal) insurance. Despite the 2014 Medicaid expansion in California, many individuals remain uninsured. Low-income women, in particular, face significant primary care access challenges due to socioeconomic status, education, and minority/disability status. This qualitative study aimed to explore the experiences of low-income women seeking and accessing primary care services following the ACA’s Medicaid expansion in California in an urban setting.

**Methods:**

In-depth, semi-structured interviews were conducted with 18 women in Northern California (2021–2022). Data analysis employed Braun and Clarke’s reflexive thematic analysis using a deductive approach. Levesque’s conceptual framework of access to healthcare guided the coding and interpretation.

**Results:**

The experiences of low-income women with primary care access post Medicaid expansion in an urban California setting were shaped by the complex interplay of individual demand-side factors and health system supply-side factors, and structural determinants. Levesque’s framework highlights how individual factors (self-efficacy, health literacy, social support, and affordable insurance) interact with health system factors (geographic accessibility, availability and accommodation of services, and provider-patient relationships) to shape low-income women’s experiences. However, Levesque’s framework could be strengthened by incorporating macro-level structural factors (socioeconomic, political factors, and health policies) as these profoundly influence healthcare access.

**Conclusions:**

These findings provide a strong foundation for policymakers and practitioners to develop multi-level policies and interventions to address the ongoing barriers that urban low-income women encounter when accessing primary care following the ACA’s Medicaid expansion. These findings are also relevant for other U.S. states and international settings that face similar challenges stemming from healthcare inequalities, including a lack of universal healthcare.

**Supplementary information:**

The online version contains supplementary material available at 10.1186/s12913-025-13853-9.

## Introduction

Achieving equitable access to primary care is a persistent global concern. In the United States (U.S.), the absence of universal healthcare coverage and long-standing income inequities have contributed to significant health inequities [1]. Recent data indicate that the U.S. performs poorly compared to primary care systems in nine other high-income countries, where more than 90% of adults in surveyed countries have a primary care provider, except Canada, Sweden, and the U.S [2]. A study of primary care access in 11 high-income countries revealed that 21% of adults overall, compared to 38% of U.S. adults, encountered multiple barriers to receiving care, while 16% of adults, compared to 18% of U.S. adults, experienced two or more barriers after reaching care, with lower-income groups encountering barriers more frequently [3]. As of 2023, life expectancy in the U.S. was 78.4 years—more than four years lower than the average among other high-income countries [4], reflecting comparatively poorer overall outcomes.

### Women’s access to healthcare in the U.S.

In the U.S., complex intersecting factors, including but not limited to age, sex and gender, race and ethnicity, immigration status, and socioeconomic factors, uniquely impact women’s access to primary care services. Intersectionality theory demonstrates how multiple competing identities, such as gender, race, ethnicity, immigration, or socioeconomic status, create intersecting and interdependent systems of disadvantage that affect women’s access to healthcare [5]. Adult women are often disproportionately affected by issues related to access to health coverage, financial costs, and discriminatory practices compared to men. For example, adult working-age women on average have lower incomes, are more likely than men to be eligible for Medicaid, less likely to be uninsured [6], and more likely to have experienced difficulties paying medical bills over the last year [7].

### The Affordable Care Act’s (ACA) role in expanding women’s access to Medicaid

Between 2010 and 2019, the ACA led to over 10 million adult women (19–64) and 7 million women of reproductive age (15–44) obtaining health coverage [8]. Before the ACA, Medicaid coverage was restricted to women who were very low income, pregnant, had children under 18 years, had a disability status, or were older than 64 years [6]. To date, ACA’s Medicaid expansion provisions have been adopted by 41 states (including the District of Columbia). The ACA provisions adopted by participating states expanded health coverage to many previously ineligible women through several mechanisms, including the expansion of Medicaid eligibility to low-income individuals (those earning below 138% of the federal poverty level), the creation of state and federal Health Insurance Marketplaces, and the introduction of premium tax credits to help individuals and small businesses purchase affordable insurance [6]. In response to the COVID-19 pandemic, the Families First Coronavirus Response Act of 2020, which included a Medicaid program requirement that recipients receive continuous coverage through the end of the COVID-19 Public Health Emergency, was enacted to reduce coverage disruptions (known as “churning”) [9]. Eligibility for Medicaid in states that did not adopt Medicaid expansion varies widely, as do coverage provisions. For example, adults without children, regardless of their income, are not eligible for Medicaid in all non-expansion states, except Wisconsin [10]. However, despite its expanded provisions, the ACA has not been an unqualified success. Among the 97.5 million women (19–64 years old) living in the U.S., 10% were still uninsured in 2023 [11]. As of 2022, 11% of women 18 years or older reported not having a healthcare provider [12].

Since the ACA, few qualitative or mixed-methods studies have explored women’s perspectives on facilitators and barriers to accessing primary care. Qualitative studies exploring women’s experiences with healthcare access post-ACA have focused on vulnerable populations, including pregnant, disabled, or older women [13], homeless women [14], immigrant or refugee women [15–20], and women receiving reproductive health services [13, 15, 21–23] in different U.S. settings. As there is a dearth of qualitative research on the perspectives of low-income women regarding access in the context of the ACA, we conducted a qualitative study to explore low-income women’s experiences seeking and using primary care services following the ACA’s Medicaid expansion in urban California, applying Levesque’s patient-centred access framework.

## Materials and methods

This qualitative study applied a reflexive thematic analysis approach, which aligns with a constructionist approach that incorporates critical framing of data, language, and meaning [24]. This approach allowed for an in-depth exploration of low-income women’s experiences accessing healthcare within a specific social context. Ethical approval for this study was granted by the Institutional Review Boards of the authors’ affiliated institutions.

### Research design

#### Population and sampling

Semi-structured interviews with 18 women facilitated in-depth personal narratives of their experiences accessing primary care services. Women were recruited from several affordable housing organisations that provide permanent housing to eligible low-income individuals or families. Women (18–64 years) who had accessed primary care services at any time following the ACA’s Medicaid expansion in California in 2014 were eligible for inclusion. The inclusion criteria did not require continuous insurance coverage, which allowed us to capture women’s experiences concerning periods of uninsurance and any subsequent challenges re-accessing care. Purposive, nonprobability sampling was used because it supports the transferability of findings to other settings [25]. Maximum variation sampling was employed to capture a wide range of perspectives on primary care access among low-income women of differing ages, races and ethnicities, educational levels, employment statuses, and relationship statuses [26]. Data collection was discontinued after we determined that sufficient in-depth rich data had been collected to address the study’s research questions, and thematic saturation had occurred. Braun and Clarke (2013) suggest 10 to 20 participants is a sufficient sample size for thematic analysis in a medium-sized study.

Study recruitment occurred between October 2021 and July 2022. Information about the research study was disseminated to potential participants through flyers, informational emails or texts sent by participating agencies to site residents, or through outreach at onsite food pantry events. Eighteen (49%) of the 37 women who showed initial interest were interviewed, 6 (16%) were ineligible, 4 (11%) refused, and 9 (24%) failed to respond to follow-up. Interviewed women were invited to share study information with other eligible women. Interested women contacted the Principal Investigator (first author) through a designated phone number or work email address. Eligibility was determined using a recruitment script. Interested women were emailed the informed consent form to review. Women provided written or verbal consent or signed consent forms electronically. Each woman had the opportunity to ask questions about the study before being interviewed.

#### Data collection

A semi-structured interview guide was piloted. The topics explored included the type of primary care provider, location of primary care services, insurance coverage, general health, behaviours regarding healthcare seeking, positive and negative experiences with primary care services, and unmet needs. After three interviews, additional questions were added to elicit information about experiences with discrimination in healthcare settings, social support, and treatment adherence, before finalizing the interview guide [Additional file 1]. The first author interviewed eligible women in person, by telephone, or using secure video conference software to ensure equitable access to the study. As all interviews were conducted during the COVID-19 pandemic, different interview modes were consistently offered throughout the recruitment period based on participant preference. Overall, 61% [11] interviews were conducted using Zoom, 22% [4] were conducted in person, and 17% [3] were conducted by phone. The recorded interviews averaged 65 minutes (36 to 88 minutes). Women also completed a short sociodemographic survey [Additional file 2]. After interview completion, a short debriefing process occurred, and women were offered a list of local mental health resources. All research participants were assigned pseudonyms to protect their anonymity. All women received a $25 gift card of their choice for their time and effort.

#### Theoretical framework: Levesque’s conceptual framework of access to healthcare

**Fig. 1:**
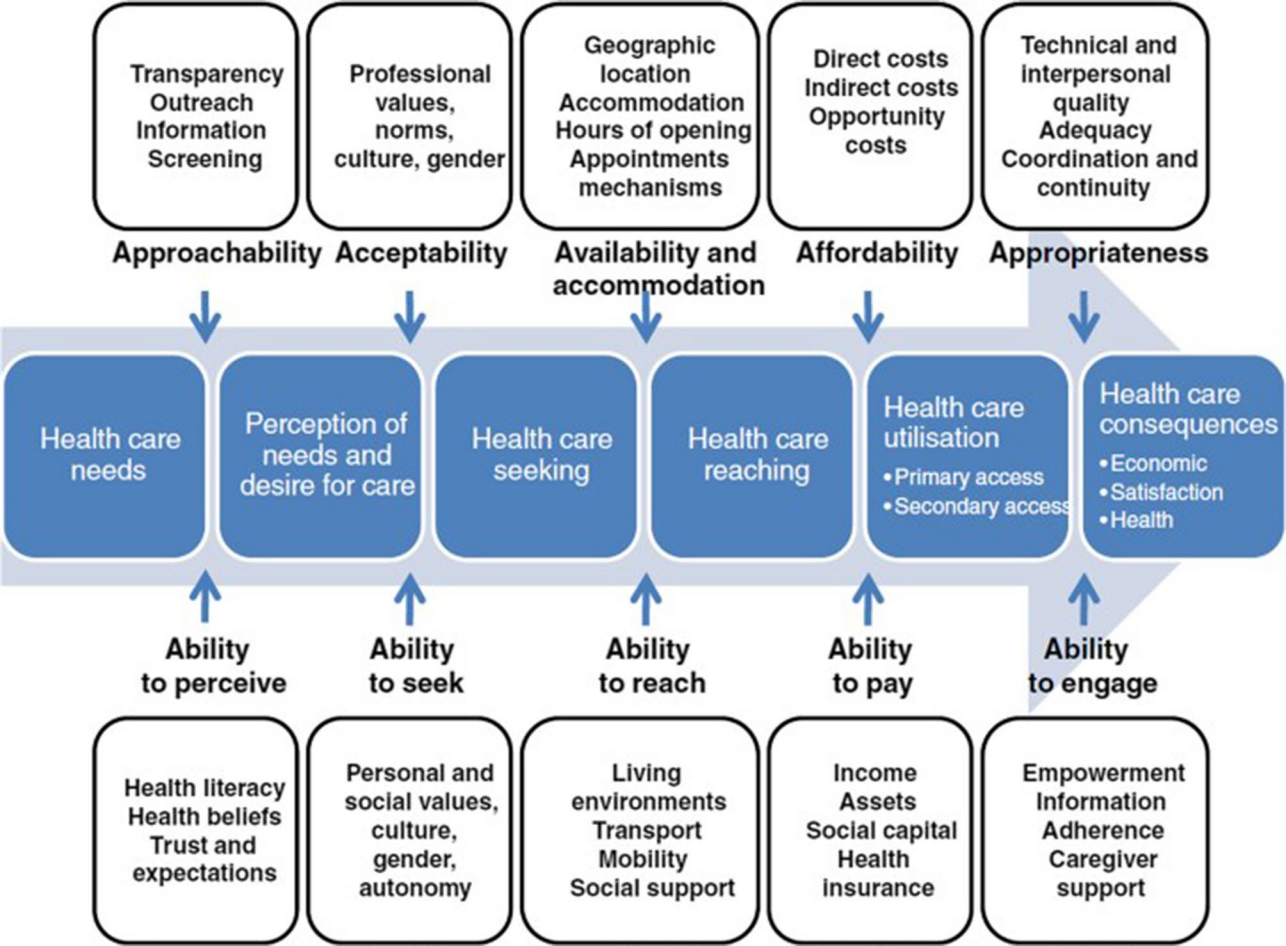
Levesque’s conceptual framework of access to healthcare. Reproduced from [27] under the Creative Commons Attribution 2.0 International License (CC BY 2.0), with kind permission from Jean-Frédéric Levesque

Levesque’s framework defines healthcare access as the interaction between individual or population demand-side factors and health system supply-side factors [27]. Adopting a patient-centred approach, the framework portrays a linear trajectory from seeking, reaching, and using healthcare services to health outcomes [27]. Demand-side factors are characterized by five dimensions—the ability of individuals to perceive, seek, reach, pay, and engage, which interact with five supply-side factors, including approachability, acceptability, availability, and accommodation, affordability, and appropriateness (Fig. 1).

Levesque’s conceptual framework of access was chosen to guide the analysis for several reasons. Based on earlier frameworks of access, the framework provides a solid foundation and logical structure for exploring multiple demand- and supply-side dimensions associated with access [27]. Embracing a person-centred focus, Levesque’s framework is a good fit for understanding women’s thoughts and perceptions about their healthcare needs, seeking and use of health services, and associated health outcomes [27]. The framework is flexible and has been applied extensively in quantitative, qualitative, and mixed-methods studies exploring diverse populations’ experiences with healthcare access in high-, middle-, and low-income country settings [28]. Reported advantages of Levesque’s framework include the evaluation of dynamic and multifaceted processes of access associated with individuals, populations, and health systems [28].

#### Data analysis

A deductive approach was applied using Levesque’s framework as an interpretive lens to explore semantic (explicit) as well as any discerned latent (implicit or deeper) meanings [29] and “patterns of shared meaning” in the dataset [30]. Emerging themes beyond the scope of Levesque’s framework were developed inductively and are reported elsewhere [31]. A unique feature of Braun and Clarke’s reflexive thematic analysis is the flexibility to apply both deductive and inductive approaches in a complementary fashion [32, 33]. Braun and Clarke’s six-stage iterative process guided the thematic analysis [33, 34]. Levesque’s framework was chosen because it is compatible with the study’s constructionist approach and epistemology, which assumes that women’s healthcare-seeking behaviours are shaped by individual life experiences embedded within a specific socioeconomic-cultural context. The first author coded the data and analysed the findings according to the individual-level demand-side and health system-related supply-side dimensions outlined in Levesque’s framework [27]. The coding tree was organised according to the ten dimensions and additional sub-dimensions of Levesque’s conceptual framework [Additional file 3]. NVivo 12 software (QSR International) was used to organise and code the data. The second and third authors guided the data analysis process.

#### Positionality and rigor

Reflexivity relies on researchers’ engagement with, and deep reflection on the data, recognition and acknowledgement of researchers’ subjectivity, and transparency on how theory impacts analysis [30]. The cultural, personal, and social background and imbibed values, beliefs, and understandings about the research topic inevitably acted as a lens influencing the researchers’ interpretation of women’s narratives. Reflexive practices, including writing field notes after each interview and annotations of research transcripts and memos, mitigated the potential for bias. Study rigour was ensured by cross-checking transcripts against interview recordings at least twice, and adherence to a detailed study protocol (credibility and dependability). In-depth interviews with women (credibility), using appropriate terms (dependability), and thick and substantive descriptions of women’s narratives (transferability) enhanced the accuracy of findings. Using a clear coding schema, field notes of interviews, annotation of interview transcripts, and research memos (confirmability) ensured methodological rigour and guided the analysis.

## Results

Women ranged from 24 to 63 years (mean = 45.8 years). Ten women identified as Black, four as Latina, one as Asian American, one as White, and two as Other. Most participants (*n* = 14) had Medi-Cal coverage, two had Dual Medi-Cal/Medicare, one had an employer-sponsored insurance, and one was covered through a parent’s Covered California plan. Table 1 summarises the key sociodemographic characteristics of the research participants.

**Table 1:** Sociodemographic characteristics of low-income women (*n* = 18)

Characteristics	n (%)*
**Age (years)**	
18–29	3 (17)
30–39	4 (22)
40–49	3 (17)
50–59	4 (22)
60–64	4 (22)
**Gender**	
Female	18 (100)
**Race/ethnicity**	
White	1 (6)
Black	10 (56)
Latina	4 (22)
Asian-American	1 (6)
Other**	2 (11)
**Relationship status**	
Single	7 (39)
Widowed	3 (17)
Divorced	6 (33)
Separated	2 (11)
**Current employment status**	
Full-time or part-time employment	6 (33)
Unemployed	6 (33)
Unable to work (disability)	6 (33)
**Education level**	
Some high school or high school	6 (33)
Some college or associate degree	9 (50)
Bachelor’s degree	3 (17)
Annual household income***	
$20,000 or less	9 (50)
$20,001–$40,000	5 (28)
$40,001–$60,000	2 (11)
$60,001–$80,000	1 (6)
Prefer not to say	1 (6)
**Country of birth**	
United States	16 (89)
Foreign born	2 (11)
**Type of insurance**	
Medi-Cal	14 (78)
Dual Medi-Cal/Medicare	2 (11)
Employer-sponsored plan	1 (6)
Covered California plan	1 (6)

*Percentages may not total 100 due to rounding to the nearest whole number

**Two women self-identified as Other (one reported South Asian immigrant, one declined to specify)

***Household size ranged from one to five persons

Table 2 summarises factors that impacted women’s experiences with primary care based on the demand-side and supply-side dimensions outlined in Levesque’s original framework. Low-income women’s access to primary care was shaped by the complex interplay of demand-side, supply-side, and structural factors as outlined in Levesque’s framework. Demand-side dimensions influencing access included women’s perceptions (health needs, motivation, self-efficacy) and practical barriers (insurance, location, safety, transport, and past experiences with health systems). Supply-side factors influenced access via approachability (e.g., primary care providers as gatekeepers), acceptability (social and cultural factors), continuity, availability, and accommodation of services (e.g., scheduling and wait times). Drivers of appropriate care also depended on provider responsiveness and patient-provider relationships. These individual and systematic factors ultimately acted as facilitators or barriers for low-income women seeking care.

**Table 2:** Factors that impact low-income women’s access to primary care services according to Levesque’s dimensions

Levesque’s dimensions	Demand-side dimensions	Supply-side dimensions
**Ability to perceive/** **Approachability**	Women’s perceptions of their health status and their perceptions regarding the need for healthcare services, level of health literacy, provider trust, and previous experiences with healthcare systems and providers influenced their use of primary care services.	Access to a primary care provider who functioned as a gatekeeper to specialty services was a key aspect of approachability. Receipt of information about scheduled check-ups and preventive health screenings fostered healthcare seeking. Transparency regarding the cost of services was important to women.
**Ability to seek/** **Acceptability**	The ability to seek healthcare was influenced by the level of personal autonomy or resourcefulness. A sense of self-efficacy and resiliency fostered women’s ability to seek health information and navigate access to health-related services.	The acceptability of services was often related to ongoing relationships with a trusted provider or whether the providers were of the same gender. Certain preventive health services, such as breast, cervical, or colorectal cancer screenings, were not always acceptable due to perceived discomfort or invasiveness.
**Ability to reach/** **Availability and accommodation**	The ability to reach services was impacted by the availability of transportation, social support, and the location of services. For example, women were more reluctant to attend primary care clinics in run-down neighborhoods where people were living on the streets or openly engaging in drug use. Some women relied heavily on social support, while others did not.	Women generally lived in close geographic proximity to healthcare services, which facilitated access. Health services that accommodate women's needs for flexibility included convenient scheduling mechanisms, short appointment wait times, and virtual consultations. Some women experienced scheduling delays of several months, especially in publicly funded health services.
**Ability to pay/** **Affordability**	Access to insurance, such as Medi-Cal, ensured low-income women could pay for health services.	Costs associated with healthcare were typically affordable with low co-pays for office visits, low costs for prescriptions, transportation, and childcare.
**Ability to engage/** **Appropriateness**	Women with chronic diseases were motivated to engage with treatment. Younger, healthier women often did not obtain regular check-ups or preventive healthcare. Poor provider communication, unresponsiveness to health needs, and perceived provider discrimination were barriers to access.	The appropriateness and quality of technical care and satisfaction with care depended on the provider and healthcare facilities. Most women reported supportive interactions with their providers; however, some narrated negative interactions with providers who ignored or discounted their concerns.

### Ability to perceive and approachability

Health beliefs, literacy, knowledge, trust, and expectations shape individual perceptions of their healthcare needs [27] and fuel the search for acute, chronic, and preventive services. Women's health-seeking behaviours were often motivated by a desire to stay healthy or take care of themselves or their families. For example, Ishani (a South Asian immigrant), recognized the importance of obtaining regular care for her autoimmune disease: *“So I do get like blood tests regularly … I am in contact with my doctor, receiving care fair amount of time, multiple times a year.”*

Most of the women underwent routine cervical and breast cancer screenings. However, adherence to colorectal cancer screening among the eight eligible women in our sample, according to current U.S. Preventive Services Task Force guidelines [35], was mixed. Two had undergone colonoscopies, three had received fecal-occult blood tests, and three had not been screened (one was not offered screening). For these women, access challenges were common and included difficulties finding a primary care provider, a usual source of care, and a lack of health information. Significantly, prior negative experiences with providers could diminish women’s trust and expectations, making them less likely to seek subsequent care.

Approachability—a characteristic of health systems that ensures individuals can readily identify and access information about available health services [27]—was generally promoted by the women’s primary care providers or place of usual care. This included information on services, treatments, and reminders for recommended preventive health screenings. While healthcare costs were often transparent, women occasionally reported receiving unexpected and costly bills. For instance, Ellie (Black) received a costly bill for blood tests following fertility services. She explained her frustration with the lack of disclosure: *“Insurance didn’t cover all of it. So, what my insurance didn’t cover, I have to pay, and I didn’t know that. If I would have [known] that, I wouldn’t have said ‘Okay, that’s fine. Let’s do them blood tests.’ You know, like I have a lot of bills accumulated, and that don’t make it no better.”* Targeted outreach could facilitate women’s access to specialized care. For example, Madeline, diagnosed with Hepatitis C, had a history of substance use disorder, and successfully accessed treatment after seeing a flyer about Hepatitis C treatment services while visiting a needle exchange programme.

### Ability to seek and acceptability

Personal autonomy, including the ability to access information and explore different healthcare options, often interacts with cultural, gender, or social factors, impacting individuals’ ability to seek care [27]. Carmelia (Latina) demonstrated agency when she proactively sought family planning services from a local clinic after losing her Medi-Cal coverage upon turning 18: “*So I needed like birth control, so I went to [Clinic].”* Later, when she got pregnant, Carmelita again exercised autonomy by researching insurance options and successfully signed up for emergency Medi-Cal at a local hospital.

In contrast, other women found it challenging to exercise personal autonomy, such as obtaining information about treatment options. For example, Lyonesse, a young mother of several children, asked her provider about effective birth control, only to be met with a recommendation for permanent sterilization: “*You should just get your tubes tied, so you don’t have any more babies … So, I felt like kind of coerced, like [he] put that idea in my mind ... I didn’t want that in my mind. I needed help, just regular conversations on something that’s going to work for me.*” Based on this recommendation, Lyonesse underwent tubal ligation, and reported she felt forced into a medical decision she was uncomfortable with.

Acceptability relies on the cultural fit of services, provider characteristics (gender, race-ethnicity, language), and professional norms [27]. Three women reported a strong preference for female providers when receiving reproductive or sexual healthcare due to concerns about comfort and safety. Ishani (a young South Asian immigrant), who had never had a pap smear, stated: *“If it’s possible, yeah, I would prefer a woman.”* Gender preference was strongly emphasized by Phoebe (Black), who had experienced an inappropriate physical examination by a male provider. She declared when switching to a new provider: *“I told them it can’t be a guy. It got to be a woman.”* Ultimately, while physician-patient gender concordance mattered to some, an established, trusted relationship was the cornerstone of acceptable health services for most.

### Ability to reach and availability and accommodation

The ability to reach healthcare is affected by factors such as personal mobility, living environment, occupational flexibility, social support, and transportation [27]. These women primarily relied on public transportation (buses, trams, walking) or non-private alternatives (cars, taxis, Ubers, paratransit) to reach health facilities. Occupational flexibility was not a significant barrier, as most women were either working part-time, unemployed, or not working due to disability.

Social support, conceptualised as logistical support (e.g., childcare or transportation) and psychosocial assistance, was often provided to participating women by family and friends. However, due to physical or mental health disabilities or a lack of social networks, some women required professional support (e.g., case managers, in-home social support workers, or social workers) to navigate access. River (Black) emphasized the importance of social worker-initiated assistance to navigate access: *“Signed me up for paratransit. Yeah, if I need something like that or in-home support, they signed me up.”* While some women lacked social support due to a lack of social ties or close family networks, others were very self-sufficient and intentionally avoided seeking help from family or friends. Ruby asserted her independence: “*Yeah, I don’t need no support. Yeah, I handle doing my business. Yeah, I do it on my own.*” Ellie, however, revealed challenges associated with this stance: “*I mean, maybe if I ask, but I’m not the type to, really. If I need it, I’ll struggle. That’s just me.”*

Availability and accommodation include the geographic location of services, hours of operation, and appointment mechanisms [27]. Most women benefited from close geographic proximity to their clinics (Jasmine recalled, *“It’s only three blocks that way … it’s walking distance, and I like where it’s at now*”), and had scheduling flexibility as 83% were either working part-time, unemployed, or had a disability. However, full-time workers such as Delilah (Black) struggled with accommodation. As she explained, *“I’m one of the essential workers. So, it’s hard to get time to take off to go to a doctor’s appointment because I have to let ‘em know three weeks in advance.”* For women like Delilah, telehealth consultations introduced during the COVID-19 pandemic were a convenient way of overcoming scheduling barriers.

Wait times were typically short, with most women seen on time or within 10 minutes. However, using publicly funded healthcare facilities sometimes led to longer waits. For example, Carmelita recalled waiting, “*… maybe like roughly 30 minutes, usually 45 minutes … to see the doctor.*” While scheduling appointments by phone, online, or in person was generally easy, health system failures created barriers for some women. Specifically, two women reported difficulties due to clinic employees failing to answer or return calls. Phoenix expressed frustration with callback issues: “*Getting someone to call you back is the issue I have with them … you have to walk in. You know it’s just a hassle*.”

Short, rushed encounters with providers often left women feeling frustrated, ignored, or excluded from medical decision-making processes. As appointment times rarely accommodated all health concerns, some women felt their needs were unmet. Trinity voiced this fear: *“I feel like if I have a list of concerns, which I usually do, then maybe I have to pick the three most important concerns, and then I deal with the others later.”* Almost all women were able to schedule an appointment within a few days or weeks; however, some experienced much longer wait times. Three women, who typically received care from publicly funded clinics, reported extended wait times of several months. Ruby captured this concern, “*So if you call for your check-up … you might have to wait 3 months to get an appointment.*” These extended wait times were likely due to a lack of providers and other resources.

Primary care providers acted as gatekeepers, providing women with necessary specialist referrals when needed. While most were satisfied with this process, a few women experienced significant delays in obtaining referrals, especially for mental health services. Lyonesse, enrolled in a Health Maintenance Organisation, waited months for a mental health specialist referral, and noted the critical system failure: “*The only one I would have a problem with is the mental health … they’re really dropping the ball on that …”* The failure of the provider to supply the necessary referral led to her being unable to obtain mental health services.

### Ability to pay and affordability

The ability to pay for healthcare is determined by financial capability (e.g., income, savings, and health insurance coverage) [27]. All participating women were employed in low-paying jobs or relied on fixed incomes and had health coverage, including Medi-Cal (California’s state-based version of Medicaid), dual Medi-Cal/Medicare, employer insurance, or Covered California (a state-run health insurance marketplace where individuals, families, and small businesses can purchase private health insurance plans). However, four women (three Latina and one Black woman) reported periods of uninsurance due to circumstances such as aging out of Medi-Cal coverage, unemployment or part-time employment, or college enrollment. Such episodes with no coverage and high costs of care often led to delays or non-receipt of healthcare services. For example, Desiree reported delaying prenatal care until the final trimester because she lacked insurance coverage and could not pay for services.

Affordability refers to the ability of the health system (including insurance, providers, and government) to manage patient costs, and encompasses both direct and indirect costs, as well as opportunity costs [27]. Health system structures presented barriers to affordability through several mechanisms related to direct, indirect, and opportunity costs. Women enrolled in Medi-Cal typically received free medical services or had minimal copays for office visits or prescriptions. However, prescription coverage was not always reliable, as women occasionally reported being billed for expensive prescription copays they could not afford. For example, two women on Medi-Cal received costly bills for medications that caused significant financial stress until their insurance plans eventually waived the charges. The design of some insurance plans, such as employer plans, posed barriers that resulted in one woman avoiding utilising her employer’s health plan for 18 months. Because of high costs, Desiree avoided seeking care: “*I would try not to go to the doctors because the co-pays were actually pretty expensive.*” Health systems did not systematically address indirect costs, as women typically had to rely on informal childcare or low-cost transportation options, such as buses, rides from family or friends to minimize costs; however, some women were provided with paratransit services or paid caregivers who could take them to appointments. However, some health facilities addressed opportunity costs by offering extended hours of services, which enabled full-time employed women to attend appointments.

### Ability to engage and appropriateness

Engagement with healthcare is the individual’s ability and motivation to engage in decision-making about preventive care or treatment [27]. We found that while women with chronic diseases typically adhered to provider appointments and prescribed treatments, younger, healthier women often engaged more sporadically, seeking care only for acute illnesses or pregnancy-related care. For example, Desiree, who was in her twenties, noted she had a usual source of care but lacked an assigned primary care provider, and confessed, “*I do not get health screenings. I haven’t gotten one in quite a while.*” Younger women’s sporadic engagement with care intensified during the COVID-19 pandemic due to reasons such as fear of contracting the COVID-19 virus, restrictive health facility protocols, or not prioritizing preventive care. Women like Destiny, recalled avoiding recent provider visits—“*I have not been since the COVID. The only time I’ve been up to [Hospital] was to get tested to make sure that I don’t have COVID.*”

Participants demonstrated engagement through seeking health information and proactive decision-making to achieve desired health outcomes. Knowing how to access healthcare information empowered some women to make informed choices about insurance, healthcare options, and treatment adherence. For instance, when Ellie’s provider discouraged her from having a child and recommended adoption, she proactively secured a referral to an obstetrician for conception services, demonstrating self-advocacy to achieve her personal health goals. Positive provider relationships encouraged mutual understanding and shared decision-making about healthcare options, further promoting engagement.

Appropriateness refers to the fit between individuals’ needs and health services, encompassing interpersonal and technical quality, timeliness, coordination, and continuity of care [27]. The women who developed long-term trusting relationships with their providers particularly valued continuity of care. River reflected on her preference for family-centred care: “*I just liked that he was the doctor to me, my mom, my brother, my son*.” For women like Talia who had several chronic diseases, appropriateness meant feeling known and personally cared for: “*She’s [primary care provider] nice. She conversates with me. She laughs with me; she jokes with me. She makes sure she makes me feel good when I come in here ‘cause she knows all of these sicknesses that I have.”*

While the women mostly felt their providers were technically proficient, a few reported incidents of poor quality or inappropriate services. For example, Carmelita felt discriminated against after a physician mistakenly assumed that she was homeless and refused to examine a skin rash, prompting her to seek care elsewhere. The quality of care coordination differed by facility and insurance plan. For older women with multiple chronic diseases, continuity of primary care services and the provider’s coordinating role were essential. Strong relationships with primary care providers that actively managed complex healthcare needs encouraged women to consistently engage with care, which may have led to better chronic disease management.

## Discussion

Access to primary care for our sample of low-income women, who were predominantly Medi-Cal beneficiaries, was driven by a complex interplay of facilitators and persistent barriers following the ACA’s expansion of Medicaid in California. In the context of Medicaid expansion, these low-income Californian women continued to encounter significant barriers to primary care access, including coverage gaps, complex navigation challenges, long appointment wait times, and discrimination in healthcare settings. These persistent barriers indicate that Medicaid expansion alone is insufficient to achieve equitable access without addressing provider- and system-level structural factors that influence the quality and continuity of care.

### Healthcare needs and perceptions about the need for healthcare

Participating women were more likely to prioritize healthcare needs if they had acute symptoms that required immediate attention. These findings are consistent with prior U.S. studies that reported homeless women only sought medical attention when their symptoms were severe [36, 37]. Compared to previous studies that demonstrate family and work responsibilities impede access for immigrant Latinas [18], women receiving reproductive services [21], and homeless women [36, 38], the majority of the women in our sample reported few competing needs.

Health literacy—the ability to “find, understand, and use information and services” [39]—is a key component of access. Challenges reported by the low-income women in our study, such as difficulty understanding and navigating complex health systems, are consistent with the barriers often related to functional health literacy. However, some women countered this by proactively seeking information from providers on conditions and treatment options. These proactive women tended to be younger or have higher levels of education, findings which are consistent with earlier studies that link higher educational attainment with higher levels of health literacy [40–42].

### Healthcare-seeking behaviours and reaching primary care

Health system factors that promoted participating women’s access to primary care included convenient scheduling mechanisms, flexible open hours, and close geographic proximity to clinics. This finding is consistent with a study of low-income urban women that showed convenient scheduling and short wait times improved access to prenatal care [22]. While most women could access reliable and affordable transportation, some reported occasional challenges. Other U.S.-based studies have also found that inadequate transportation hinders access for uninsured immigrant women [17, 18], rural women [43], and those receiving reproductive healthcare services [13, 15, 21, 23].

For some women in this study, the need for social support to navigate access to primary care was influenced by intersecting individual, familial, and cultural factors. These findings are consistent with prior research demonstrating the key role that social support from family and friends played in promoting access for immigrant women [17, 18, 44–46], and how the absence of social support can pose a significant barrier [18]. We found that for some women, professional navigation services provided a critical bridge to accessing needed care. The supportive role of care coordinators in arranging transportation and resources can help individuals overcome specific barriers to access [47].

### Healthcare utilisation and consequences

Despite ACA provisions mandating that Medicaid cover preventive care without patient cost sharing, some women enrolled in Medi-Cal were not up to date with breast cancer or colorectal cancer screenings. Only 25% of those women eligible for colorectal cancer screenings had undergone colonoscopies. Non-adherence to colon cancer screening may be linked to anxiety, inconvenience, or fear of discomfort [48]. In our sample, two younger Latinas and one Black woman reported they had not had a recent check-up due to lack of coverage or competing needs. These findings are consistent with a 2020 national survey that found low-income (64%), uninsured (41%), younger (18–25 years) (59%), and Hispanic women (67%) were less likely to have had a recent check-up in the past 2 years, compared to other groups [49]. In low-income populations, uninsured status, especially among immigrant Latina women [15, 17, 18, 50], as well as competing needs and transportation difficulties [51], are persistent barriers to primary care.

Several women reported experiences with healthcare discrimination based on racial-ethnic minority status, gender, history of mental illness, or housing status. This perceived discrimination resulted in inappropriate care and mistrust of providers. Our findings that discrimination and stigma lead to medical mistrust and delayed or non-receipt of care are supported by a systematic review [52], and several U.S. studies across different populations of vulnerable women, including immigrants [16, 53, 54], reproductive health services [23, 55], rural areas [43], and publicly insured adults [56].

Conversely, positive patient-provider relationships facilitated engagement with care. Women who reported strong relationships with their providers appeared more satisfied with their care and demonstrated better adherence to regular check-ups, preventive screening, and prescribed treatments. These findings are consistent with extensive research that demonstrates effective patient-provider communication, compassionate care, provider competency, and continuity of care promote trust and improve low-income women’s satisfaction with preventive and reproductive healthcare services [22, 55, 57].

### Study limitations

These findings are subject to several limitations, which may impact their transferability to other urban California settings or different geographic regions in the U.S. Our inclusion criteria restricted our sample to low-income women who had previously engaged with and accessed primary care at any time since California’s Medicaid expansion. Therefore, women without insurance coverage for the duration of the period under consideration were ineligible for participation in this study. As our sample consisted mainly of Black and Latina women, this meant that the healthcare experiences of women from other minority groups, such as Asian/Pacific Islander and Native Americans, are not represented. The exclusion of non-English-speaking immigrants from participation was also a limitation, as funding was not available for translation services. Our findings may also be limited as the perspectives of women living in affordable housing might not represent those of low-income women living in other settings. Offering different interview modes (Zoom, phone, and in-person) may have affected the interviewer's ability to observe non-verbal cues or establish rapport; however, using the same semi-structured interview guide with all participants minimized the impact associated with different interview modalities. Additionally, women may have provided socially desirable responses, particularly around sensitive topics, which could have distorted the findings [58]. Finally, the deductive analysis applied the dimensions outlined in Levesque’s framework, potentially restricting emerging concepts or themes. To address some of these limitations, we recommend that future qualitative research prioritize exploring barriers in more diverse populations of vulnerable women.

### Applicability of Levesque’s framework

Levesque’s framework was selected for the deductive analysis because it provides a comprehensive and multidimensional model of healthcare access and a systematic way to categorize factors that influence women’s access to care [28]. We found most dimensions, such as the ability to reach, ability to pay, affordability, appropriateness, and availability/accommodation, were easily operationalized and captured during the coding process. However, less easily definable constructs, such as acceptability and approachability, proved more difficult to measure directly [28]. Since some framework dimensions involve a complex interplay of cultural, personal, and social factors, this suggests they are better captured using qualitative methodology. While Levesque’s framework accounts for physical and social living environments, it does not consider wider macro-level factors that influence healthcare access. For example, the framework fails to account for how healthcare policies, such as the ACA, or overall funding mechanisms, affect population access [59]. We suggest the framework be enhanced by explicitly incorporating such essential macro-level structural factors—specifically, the socioeconomic and political factors that inform national or local healthcare policies.

### Implications and recommendations for policy and practice

Recommendations are structured to directly address the major barriers identified. Some recommendations extend beyond Levesque’s framework, such as suggestions for initiatives to combat implicit bias and discrimination in healthcare settings.

#### Enhancing navigation of access and health services

We found that lower-income women often require assistance with navigating better access and flexible services to address logistical barriers to primary care. To address this, policies and programmes should streamline the process involved with reaching healthcare services and offer expanded services.

**Support with patient enrollment and navigation:** Healthcare delivery systems should invest more in patient navigation services (such as those provided by case managers, patient navigators, or social workers) to assist low-income women with enrolling in coverage, locating in-network providers, scheduling appointments, and arranging transportation.

**Enhancing clinic services:** Health system-driven strategies include more efficient appointment scheduling mechanisms, accessible online health apps and portals, increased appointment availability to reduce wait times, expanded clinic hours, and telehealth services. Recent initiatives, such as California’s 2022 California Advancing and Innovating Medi-Cal (CalAIM) program, established Enhanced Care Management and Community Supports, which provide in-person care management and non-medical supports (e.g., housing, nutrition, transportation) to high-need Medi-Cal beneficiaries in managed care plans [60]. Such programs offer promising approaches to providing integrated medical, behavioural, and social services, and could be beneficial for low-income women with complex and intersecting medical and social challenges.

#### Addressing health system barriers to access

Utilisation of care was often influenced by the women’s personal health beliefs, level of health literacy, and experiences of discrimination. To ensure care is both acceptable and appropriate, health systems must address issues related to cultural competency, organisational health literacy, and systemic bias.

**Culturally Competent Care and Health Literacy:** To provide acceptable and appropriate services, strategies include cultural matching of providers with patients and using culturally appropriate materials. Healthcare services need to systematically promote strategies to enhance health literacy, such as ensuring health information is accessible and understandable, and providers must consistently provide clear patient education [61].

**Tackling discrimination:** To combat discriminatory practices in healthcare settings, it is essential to develop long-term educational strategies focused on systematically training diverse groups of healthcare providers across different healthcare settings [62]. This includes health system-mandated training of healthcare workers in cultural competency and implementing required curricula on implicit bias, early in clinician training programs [63].

## Conclusion

This qualitative study provides insight into the experiences of urban low-income women (predominantly Medi-Cal recipients) accessing primary care post-Medicaid expansion in California. Our findings emphasize that access is shaped by a dynamic interplay of demand-side individual factors (self-efficacy, health literacy, social support) and supply-side health system factors (geographic proximity, availability and accommodation, continuity and quality of provider-patient relationships). While factors like affordable coverage and available health services (e.g., flexible scheduling/telehealth visits) are key facilitators of access, barriers include disruptions in coverage, navigation difficulties, long waits/referral bottlenecks, poor provider communication, and discrimination in healthcare settings. These findings demonstrate that while Medicaid coverage is an essential component of access for low-income women, other barriers often impede access to timely and appropriate primary care.

The findings provide a foundation for policymakers and practitioners to develop multilevel programs and interventions, beyond insurance coverage, that target navigation support and linkage of low-income women, especially those with complex healthcare needs, to comprehensive and coordinated care management (e.g., CalAIM services). We further recommend extending Levesque’s framework to explicitly incorporate macro-level structural drivers (e.g., policy design and financing) to better capture determinants of equitable access and guide the design of interventions to reduce health inequities.

## Electronic supplementary material

Below is the link to the electronic supplementary material.

Supplementary Material 1

Supplementary Material 2

Supplementary Material 3

## Data Availability

The dataset generated and analysed during the current study is not publicly available due to considerations of confidentiality, but is available from the Faculty of Health and Medicine, Lancaster University, Lancaster, United Kingdom, email: rdm@lancaster.ac.uk, upon a reasonable request.

## Declarations

### Authors’ information (optional)

AG was a doctoral candidate at the Division of Health Research, Faculty of Health and Medicine, Lancaster University at the time this research was conducted, and is an Assistant Professor at the College of Health and Social Services, San Francisco State University, California, U.S. PH is a Senior Lecturer and FA is a Lecturer at the Division of Health Research, Faculty of Health and Medicine, Lancaster University, Lancaster, United Kingdom.

## Abbreviations

ACA: Affordable Care Act
CalAIM: California Advancing and Innovating Medi-Cal
U.S.: United States
