# Supplementary material for: Accessing primary care following the Affordable Care Act: a qualitative study of low-income women’s experiences in urban California
Source: BMC Health Serv Res. 2026 Feb 4;26:331. doi: 10.1186/s12913-025-13853-9 (PMC12964643; doi:10.1186/s12913-025-13853-9)
Supplement: Supplementary file 1 — Supplementary Material 1 [file 12913_2025_13853_MOESM1_ESM.docx]

**Interview Guide**

***Introduction: In California, the Affordable Care Act (ACA) was passed into law in 2010, but most of the ACA provisions went into effect in 2014, including Medicaid expansion, which expanded health coverage to more Americans below a certain income level. I am interested in your experiences since the beginning of 2014 or over the last 7 to 8 years, or so I am going to ask you questions about your experiences accessing or using primary care services for an acute or chronic health concern, preventive healthcare (such as an annual check-up, health screening) and also your experiences around seeking primary care services for these services.***

**Instructions:**

*Explore what the participant calls the provider of primary care services and/or place of usual source of care. Once the provider and/or place of usual source of care reference terminology is identified, the interviewer will use this throughout the interview. Primary care providers might be doctors, Physician’s Assistants, or Nurse Practitioners who provide care for acute or chronic health concerns or preventive healthcare such as health check-ups and screening tests (such as blood pressure checks, labs, or cancer screening tests, such as pap smears, mammograms). A usual source of care might be a doctor’s office, clinic, community health centre, hospital clinic, urgent care, or emergency room. Questions (and prompts) will be adapted or eliminated based on whether the participant covered the area of interest in sufficient detail for this study.*

Access to primary care services

1. **Usual source of care: Can you tell me where you usually get your primary care?**

***Prompts:*** *[Where do you go to get your primary care (e.g., a doctor’s office, clinic, community health centre, hospital clinic, urgent care, or emergency room)? What type of provider do you see for your primary care (e.g., is there a particular doctor, nurse, or another health professional that you see most often for primary care services)? Where have you gone for primary care services over the past 7 to 8 years or so, since the beginning of 2014? About how long has it been since you last visited a provider (e.g., doctor) for a routine check-up/annual physical exam? In the past 12 months, not counting the times you went to an emergency room, how many times did you go to your primary care provider to get care for yourself? How many times did you go to see specialists to get care for yourself?]*

1. **Health insurance:** Do you have any kind of healthcare coverage (for example, including health insurance, prepaid plans such as Health Maintenance Organisations, or government or state-funded plans such as Medi-Cal or Medicare)?

***Prompts:*** *[If yes, what type of healthcare coverage do you have? If not, where do you go to get healthcare if you need it? How do you pay for your healthcare? Since October of 2013, have you tried to get health insurance for yourself through the new federal healthcare law (e.g., through ACA’s Medicaid expansion from healthcare.gov or a state website)? If yes, can you tell me about the process? How easy or difficult was it to obtain health insurance?]*

1. **General Health: Can you tell me about your general health?**

***Prompts:*** *[How would you describe your general health? Why? Overall, how confident are you about your ability to take good care of your health? How do you take care of your health? Do you have any chronic conditions that you are currently receiving care for? If yes, what conditions are you currently receiving care from your primary care provider for (e.g., explore different types of providers who receive care for all conditions discussed)]*

1. **Preventive Healthcare:** Can you tell me about any preventive healthcare (such as health screenings) you get?

***Prompts:*** *[Have you ever had a pap smear to check for cervical cancer? If yes, when was your last pap test/smear? Have you ever had a mammogram to check for breast cancer? If yes, when was your last mammogram? Have you ever had any kind of tests to check for colorectal cancer? If yes, what type of colon screening test did you have (e.g., colonoscopy or stool tested for blood)? When was your last test to check for colon cancer? Have you ever had your glucose checked for diabetes? If yes, when was your last blood test to check for diabetes? Do you get your BP checked by your provider (for example, a doctor or nurse)? If yes, when was your BP last checked? Where do you get your blood pressure checked? Do you know your blood pressure? (e.g., a normal BP is 120/80)? [If a woman of childbearing age] Have you used family planning services in the last 7 to 8 years? If yes, what type of services have you used? Have you used prenatal care services in the last 7 to 8 years? If yes, what type of services have you used? How does your provider’s office reach out to you with appointment reminders about routine medical care (annual check-ups, preventive care screenings such as pap smears and mammograms)? Do you see other types of healthcare providers besides your primary care provider (doctor, nurse)? If yes, why do you go to see this/these provider(s)?]*

1. **Experiences with primary care services****: Tell me about your experiences visiting your primary care provider or usual source of care.**

***Prompts:*** *[Tell me about the most recent visit to your primary care provider or usual source of care. When was it? Where was it? What was the reason? What took place during this visit? Was your health problem resolved? Tell me about your interactions (experience) with the staff. Tell me about your interactions (experience) with your primary care provider?]*

1. **What do you think about the primary care services at the place where you get care?**

***Prompts:*** *[What do you think about the quality of care? Ease of (easy to get) appointments? Hours of operation? Walk-in facilities? Geographic location? Friendliness of the staff? Average waiting time? Physical environment/condition of the healthcare facility? The neighbourhood of the facility? Cost of services (e.g., direct costs such as copays, deductibles, costs; indirect costs such as transportation costs, parking, babysitting; opportunity costs such as lost wages)? Technical and interpersonal skills of provider (e.g., communication skills, interpersonal skills, competent, knowledgeable, ethical)?*

*What do you think about the quality of the information provided to you by your provider(s) about health problems, procedures, tests, labs, or treatments ordered for you? Are you able to ask your provider questions? Explain. Are you satisfied with the answers that your provider gives you? Continuity of care? Coordination of care? Cultural appropriateness of care? Language? How good a job does a healthcare facility do with providing health-related information (e.g., brochures), language services, language forms, and education brochures in (language)?]*

*Are you likely to return to your primary care provider or usual source of care for primary care services/follow-up? [Why, why not? What might make it difficult? What might make it easier? What would help you change your mind? Any suggestions about ways that the primary care services could be improved?]*

1. **Tell me about any experiences of problems or difficulties when you went to see your primary care provider.**

***Prompts:*** *[What was it about the visit that was a problem or difficult (e.g., issues with providers, staff, tests, examination)? Can you describe each problem or difficulty? Why was this a problem? How did the visit make you feel?]*

1. **Tell me about any experiences when your visit went well when you went to see your primary care provider.**

***Prompts:*** *[What was it about the visit that went well? Why did the visit go well? How did the visit make you feel?]*

1. **Unmet needs: Can you tell me about any health needs you have that have not been met?**

***Prompts:*** *[Describe how your health needs are not being met [by your primary care provider/usual source of care]? Why is it difficult to get your health needs met? How does this make you feel?]*

1. **Health-Seeking behaviour:**
   1. **What are some of the reasons you seek healthcare?**

***Prompt:*** *[What makes you seek help when you are sick?].*

- 1. **Tell me about any experiences you had where you needed help for a health problem or preventive care, and you had a problem getting the care you needed.**

***Prompts:*** *[What made it difficult to get the care you needed? Probe for the following: difficulty getting an appointment, transportation issues, mobility, not having someone go with you to the provider, too many things going on, unable to take time off work, fearful or anxious about going to the provider (e.g., didn’t want to know what was wrong), no social support, cost (direct cost, indirect cost, opportunity costs, e.g., loss of work, no insurance)? Why was it difficult? What did you do to overcome the difficulty and get the care you needed? What influenced your decision-making? How did the experience make you feel?]*

- 1. **Tell me about any experiences you have where you needed help for a health problem or preventive care, and it was easy to get the care you needed.**

***Prompts:*** *[What made it easy to get the healthcare you needed? Why was it easy to get the care? What influenced your decision-making? How did the experience make you feel?]*

1. **Discrimination: Can you tell me about any experiences with discrimination when seeking or getting primary healthcare services?**

***Prompts:*** *[What happened? How did this impact your willingness to seek primary care services? How did this impact your behaviour seeking primary care services? How did this impact your ability to get the primary care services you needed?]*

1. **Social Support: Can you tell me about any type of support you get from family members, friends, or other individuals, such as case managers and social workers, to get primary care services?**

***Prompts:*** *[Who helps support you with getting primary care services? What type of support do they provide? How does the support you receive help you to see your primary care provider/get the primary care services you need?]*

1. **Treatment/management strategies: Can you tell me how you manage any treatments and/or medications that you receive for acute or chronic conditions from your primary care provider?**

***Prompts:*** *[What information do you need to manage any conditions that you have? What helps you to adhere to your treatments/medications ordered by your provider? What support do you receive in helping you to manage your treatment/medications? Do you experience difficulties with managing your treatment/medications? If yes, why?]*

1. **Is there anything else you would like to say about your experiences accessing or using primary care services that you have not discussed already?**
