# Supplementary figures and images for: Accessing primary care following the Affordable Care Act: a qualitative study of low-income women’s experiences in urban California
Source: BMC Health Serv Res. 2026 Feb 4;26:331. doi: 10.1186/s12913-025-13853-9 (PMC12964643; doi:10.1186/s12913-025-13853-9)

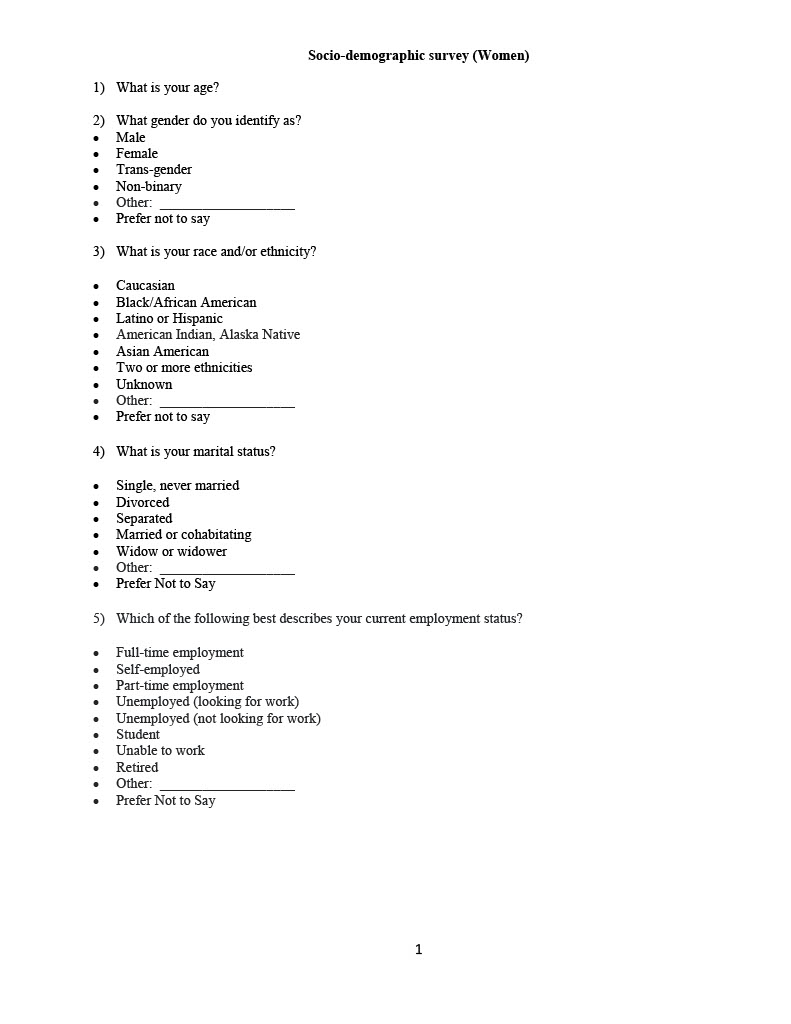


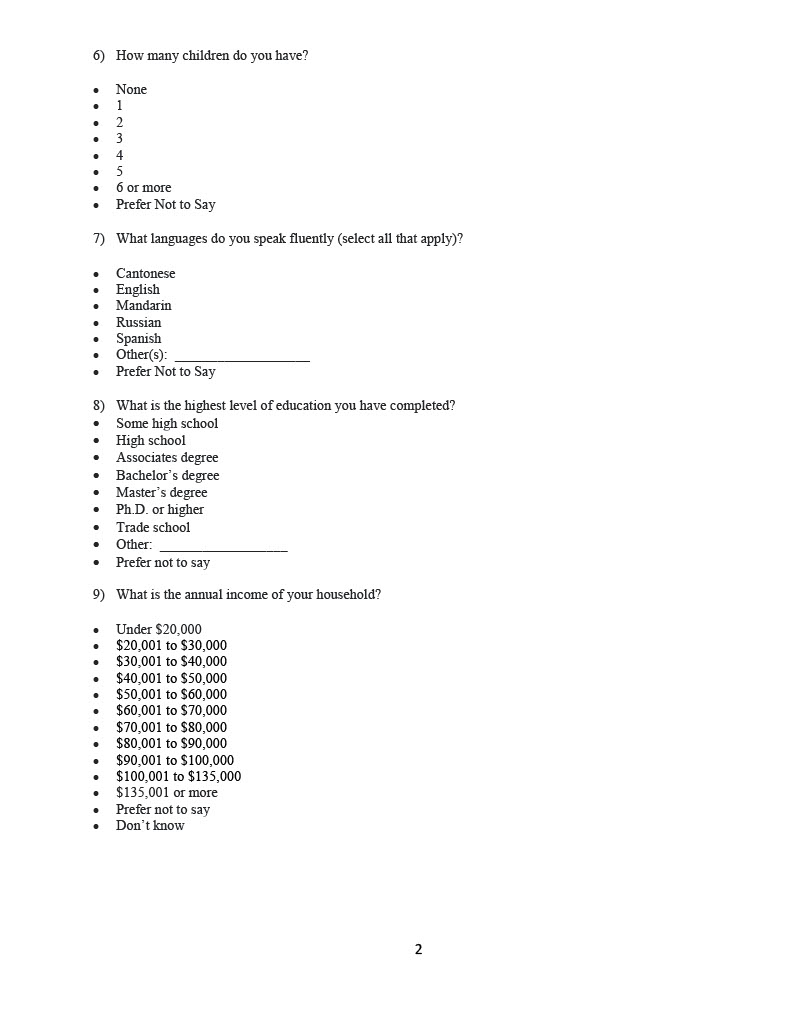


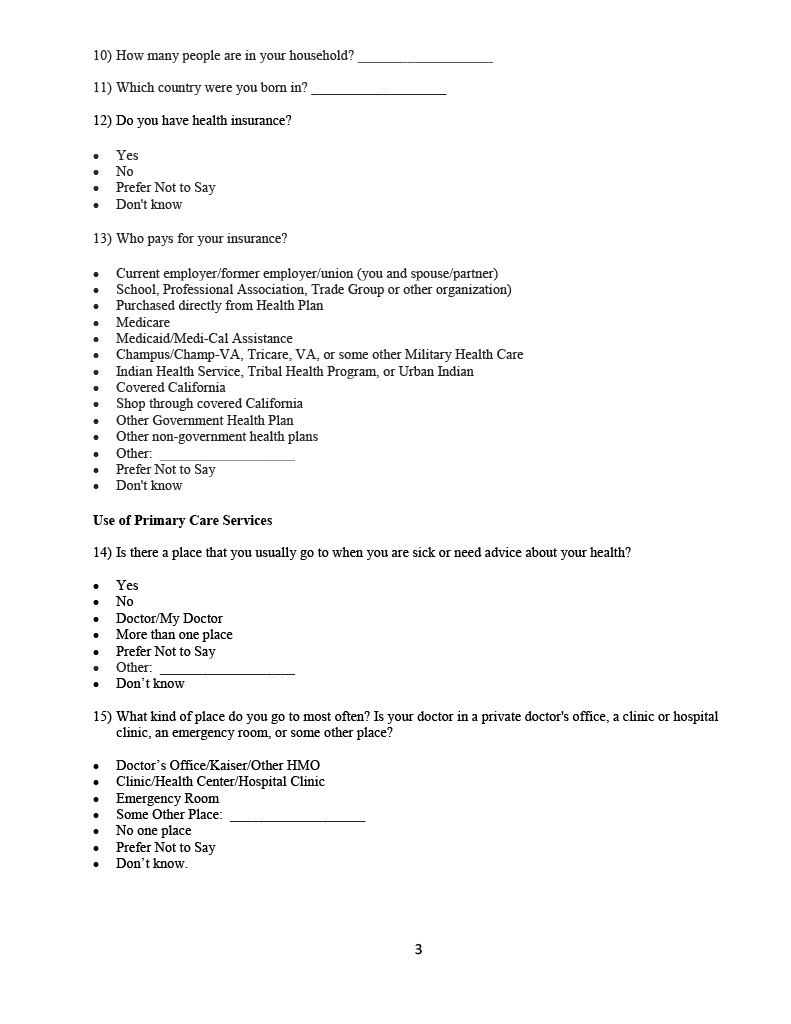

Supplement: Supplementary file 2 — Supplementary Material 2 [file 12913_2025_13853_MOESM2_ESM.docx]
