# Supplementary material for: Accessing primary care following the Affordable Care Act: a qualitative study of low-income women’s experiences in urban California
Source: BMC Health Serv Res. 2026 Feb 4;26:331. doi: 10.1186/s12913-025-13853-9 (PMC12964643; doi:10.1186/s12913-025-13853-9)
Supplement: Supplementary file 3 — Supplementary Material 3 [file 12913_2025_13853_MOESM3_ESM.docx]

**Additional file 3.** **Coding tree for deductive data analysis according to Levesque’s conceptual framework of access to healthcare**

***Accessibility of services (dimensions capturing the demand-side of determinants****)*

| **Dimensions of access** | **Components (codes)** | **Definition** |
| --- | --- | --- |
| **Ability to perceive** |  | Individuals' perceptions of their healthcare needs are shaped by their beliefs related to health and illness, health literacy, health-related knowledge, trust, and expectations. |
|  | **Health literacy** | Personal health literacy can be defined as how well individuals can locate, understand, and use health-related information and services to make health-related decisions and act either for themselves or other individuals (Centers for Disease Control and Prevention, 2022). |
|  | **Health beliefs** | Individual beliefs about health, the cause of illness, and ways to prevent and overcome illness. |
|  | **Trust and expectation** | Established trusting relationships with providers. Expectations about providers and healthcare services received. |
| **Ability to seek** | **Personal and**  **social values,**  **culture,**  **gender,**  **autonomy** | Personal, cultural, gender, and social-related values that affect an individual’s ability to seek care. |
| **Ability to reach** |  | The geographic accessibility of services, mobility, transportation, and social support that affect an individual’s ability to reach primary health services |
|  | **Living environments** | How physical and social environmental structures (e.g., housing, neighbourhood, or proximity to primary care services) affect health service use by individuals. |
|  | **Transport** | Availability of transportation, and the ability to use transportation to reach primary care services. |
|  | **Mobility** | Physical mobility and the ability to reach primary care services. |
|  | **Social support** | Availability of social support from family, friends, professional caregivers, and social services. |
| **Ability to pay** |  | The ability to pay for healthcare services is determined by financial capability, which includes not only income, savings, or loans, but also health insurance coverage with associated costs. |
|  | **Income** | Income to pay for direct and indirect costs associated with using primary care services. |
|  | **Assets** | Access to assets that can pay for healthcare costs. |
|  | **Social capital** | Networks of relationships between individuals that allow individuals access to care. |
|  | **Health Insurance** | Access to health insurance, which covers expenses for medical treatment and preventive healthcare. |
| **Ability to engage** |  | Seeking and obtaining healthcare requires individuals to engage in decision-making about preventive care or treatment, and is influenced by the ability and motivation to engage with care. |
|  | **Empowerment** | The process by which an individual is empowered to make decisions and take actions relating to healthcare. |
|  | **Information** | Individuals' ability to access and utilise information from their providers. |
|  | **Adherence** | Ability to adhere to medications and treatments. |
|  | **Caregiver support** | Need for caregiver support (e.g., from family, friends, professional caregivers, social workers). |

Source. Levesque et al. (2013)

***Accessibility of services (dimensions capturing supply-side determinants)***

| **Dimensions of access** | **Components (codes)** | **Definition** |
| --- | --- | --- |
| **Approachability** |  | Approachability means that individuals with health needs can identify health services that exist, are reachable, and can impact health outcomes. |
|  | **Transparency** | Price transparency, provision of pricing estimates for select items and services before care is delivered. |
|  | **Outreach** | Outreach activities to encourage individuals and populations to access primary care services. |
|  | **Information** | Information about services and treatments available. |
|  | **Screening** | Knowledge about recommended screenings and utilisation of screening services. |
| **Acceptability** | **Professional values, norms, culture, and gender** | Professional values of providers, norms associated with the delivery of healthcare services, cultural acceptability of healthcare services provided, and the gender of the provider. |
| **Availability and accommodation** |  | Availability and accommodation refer to geographic availability of services, accommodation of healthcare facilities (i.e., flexible hours, modes of service provision, or types of available providers), hours of opening, and appointment mechanisms. |
|  | **Geographic location** | Access to the geographic location of healthcare services facility (e.g., accessible by public transportation). |
|  | **Accommodation** | Accommodation of healthcare facilities. For example, flexible hours to accommodate work schedules, and flexibility in the provision of services (e.g., contact procedures, virtual consultations). |
|  | **Hours of opening** | The hours that services are available at healthcare facilities. |
|  | **Appointments mechanisms** | Process by which appointments are made (e.g., by phone, in-person, using an online app). |
| **Affordability** |  | Affordability emphasizes the cost of services and the ability of the healthcare delivery system to ensure that patients can manage healthcare costs. |
|  | **Direct costs** | Direct price of services (e.g., copays, out-of-pocket costs, deductibles). |
|  | **Indirect costs** | Cost related to transportation (e.g., cost of using own vehicle, taking public transportation, taxi, Uber). |
|  | **Opportunity costs** | Lost income if not working (e.g., having to take unpaid time off work to attend appointments). |
| **Appropriateness** |  | Appropriateness is the fit between individuals' needs and health services, the interpersonal and technical quality of provided services, and the timeliness with which providers assess, diagnose, and treat health problems. |
|  | **Technical and interpersonal quality** | The technical ability of providers and staff to provide primary care services and the interpersonal quality of interactions. |
|  | **Adequacy** | Appropriateness and quality of services provided. |
|  | **Coordination and continuity** | Continuity of care and coordination of services by a PCP. |

Source. Levesque et al. (2013)
